# Comparison of the Bond Strength to Titanium of Resin-Based Materials Fabricated by Additive and Subtractive Manufacturing Methods

**DOI:** 10.3390/polym18010056

**Published:** 2025-12-24

**Authors:** Asiye Yavşan, Recep Türken

**Affiliations:** Department of Prosthodontics, Faculty of Dentistry, Ordu University, Ordu 52200, Türkiye; asiyeyavsan@odu.edu.tr

**Keywords:** 3D printing, CAD/CAM, titanium, bond strength

## Abstract

This in vitro study investigated the shear bond strength (SBS) between titanium abutments and resin-based CAD/CAM restorative materials fabricated using additive (3D printing) and subtractive (milling) methods. The aim was to assess how different surface treatments—primer only, phosphoric acid etching with primer, and sandblasting with primer—affect bonding performance. A total of 120 cylindrical specimens were prepared using four CAD/CAM materials and bonded to titanium disks using dual-cure resin cement. SBS was measured following ISO 10477:2020 guidelines, and surface morphology was analyzed via scanning electron microscopy (SEM). Two-way ANOVA revealed that both the material type and surface treatment had statistically significant effects on SBS (*p* < 0.001), with a notable interaction between them. Additively manufactured materials exhibited higher SBS values compared to subtractive ones. The highest bond strength was observed in the sandblasted Saremco Crowntec group, while the lowest was in the primer-only Cerasmart group. SEM images confirmed enhanced surface roughness in sandblasted specimens, and failure mode analysis showed more cohesive and mixed failures in mechanically treated groups. These findings underscore the importance of selecting appropriate surface conditioning protocols tailored to each material type to improve bonding effectiveness in implant-supported restorations.

## 1. Introduction

Temporary restorations, whether short- or long-term, play a vital role in prosthetic dental treatment by supporting soft tissue healing, ensuring occlusal stability, and improving patient satisfaction. As their clinical use expands, the surface durability, adhesive quality, and mechanical stability of the materials become increasingly critical, especially for implant-supported cases, where the bond strength to the abutment directly affects clinical success [1].

In response, CAD/CAM technologies have transformed restorative workflows by enabling fast, precise, and reproducible fabrication of patient-specific restorations. Additive manufacturing (AM) offers advantages such as minimal material waste, shorter production times, and high design flexibility, while subtractive manufacturing (SM), with proven clinical success, remains preferred for high-strength applications [2]. Although resin-based CAD/CAM materials now support both techniques and meet functional and aesthetic demands, their bonding behavior to abutment substrates like titanium—and their response to surface treatments—remains an area requiring further investigation [3].

The success of implant-supported restorations depends largely on the biological and mechanical compatibility of the abutment material. Titanium remains the gold standard due to its excellent biocompatibility, corrosion resistance, and mechanical strength. Grade V titanium alloy, commonly used in implant abutments, offers high tensile strength and a bone-like elastic modulus that supports physiological stress distribution [4,5].

The integration of Ti-base abutment systems with CAD-CAM technology reflects digital dentistry’s progress, combining titanium’s mechanical advantages with the precision of digital workflows. However, titanium’s inert surface chemistry and low surface energy pose challenges to achieving reliable adhesion with resin-based or ceramic restorative materials [6].

Achieving durable adhesion between prosthetic materials and inert substrates like titanium requires modifications to both surface topography and chemistry. Mechanical treatments, such as aluminum oxide sandblasting, increase surface energy and promote micromechanical interlocking [7]. Alternatively, tribochemical silica coating embeds SiO_2_ particles, enabling both mechanical and chemical bonding, though some studies report lower bond strength compared to sandblasting [8].

Chemical treatments using primers aim to enhance surface reactivity. Primers containing 10-MDP (10-Methacryloyloxydecyl dihydrogen phosphate), like Alloy Primer (Kuraray Noritake Dental Inc., Tokyo, Japan) and Monobond Plus (Ivoclar Vivadent AG, Schaan, Liechtenstein), form strong chemical bonds with titanium. Alloy Primer includes VBATDT [6-(4-vinylbenzyl-N-propyl)amino-1,3,5-triazine-2,4-dithiol] for enhanced metal affinity, while Monobond Plus combines MDP, silane, and disulphide acrylate, making it suitable for hybrid surfaces [9,10,11]. Studies suggest that combining mechanical and chemical treatments yields superior bond strength compared to either method alone [12,13,14].

The biomechanical properties, surface energies, and adhesive interactions of resin-based materials produced via additive and subtractive CAD/CAM methods are key factors in the long-term success of prosthetic restorations [15]. A comprehensive understanding of the factors influencing bond strength between these restorative materials and titanium abutments, especially when subjected to various surface treatments, is essential for enhancing clinical results and preventing complications such as debonding or fracture. Although significant research has focused on the bonding characteristics of titanium abutments with restorative materials, a universally accepted protocol for the cementation of Ti-base abutments with different restorative materials and cementation agents has not yet been established [16]. Küçükekenci et al. [17] reported higher SBS for AM resin-based crowns on titanium compared with SM materials, the present study expands on these findings by evaluating two next-generation resin-based CAD/CAM materials under both additive and subtractive manufacturing methods and by directly comparing surface treatments (primer, sandblasting, acid) to identify material- and process-specific optimization strategies.

This study aims to evaluate the bond strength between new-generation resin-based CAD/CAM materials and titanium substructures produced via AM and SM methods. Understanding how these production techniques affect bonding to titanium abutments is crucial for appropriate material and method selection in clinical practice. Additionally, the study investigates the influence of various mechanical and chemical surface treatment protocols on bond strength.

The null hypothesis of the study is that different production methods (AM and SM) and applied surface treatment protocols (sandblasting, acid-etching, primers containing MDP) will not produce a significant difference in the bond strength of resin-based restorative materials to titanium.

## 2. Materials and Methods

A total of 120 cylindrical restorative specimens (Ø3 × 4 mm) were fabricated using both AM and SM methods, including two additively manufactured resin materials—Saremco Crowntec [SC] (Saremco Dental AG, Rebstein, Switzerland) and VarseoSmile Crown Plus [VS] (BEGO, Bremen, Germany)—as well as two subtractively manufactured materials—Tetric CAD [TC] (Ivoclar Vivadent AG, Liechtenstein) and Cerasmart [CS] (GC Corporation, Tokyo, Japan) (Table 1). The cylindrical specimen design was created using Exocad DentalCAD design software (Exocad GmbH, Darmstadt, Germany; version 3.1 Rijeka) in STL (Standard Tessellation Language) format. The fabrication process utilised either milling (SM) or 3D printing (AM) technologies, depending on the material type.

**Figure 1:**
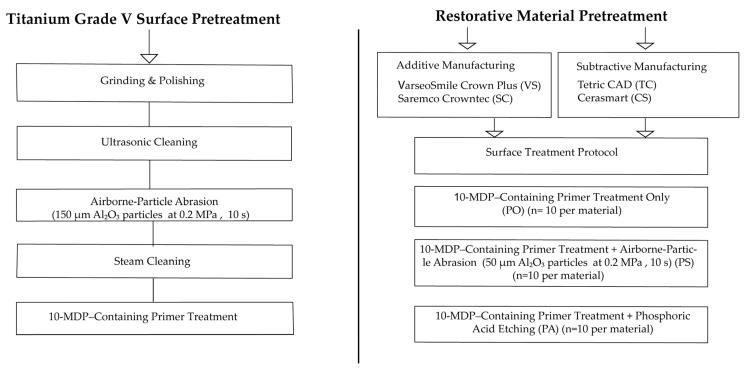
Schematic illustration of the experimental workflow.

SC specimens were produced using the Nextdent 5100 3D printer (3D Systems, Rock Hill, SC, USA), while VS specimens were fabricated using the SprintRay Pro 95s 3D printer (SprintRay Inc., Los Angeles, CA, USA). For both materials, the layer thickness was set to 50 µm, and the specimens were oriented horizontally (0°) on the build platform. This standard orientation and layer thickness were selected in accordance with the manufacturers’ recommendations and to obtain a clinically relevant printing protocol with consistent build accuracy and surface quality [2,18]. Support structures were generated automatically and removed manually after the printing process. Following fabrication, all AM specimens underwent ultrasonic cleaning to eliminate uncured resin residues. The specimens were immersed in 99% isopropyl alcohol and cleaned using an ultrasonic bath (JP-4820; Skymen Heatable, Shenzhen, China) in two stages: 3 min of pre-cleaning followed by 3 min of main cleaning. After cleaning, specimens were air-dried. Subsequently, post-curing was performed according to the manufacturers’ protocols to eliminate residual monomers and enhance the mechanical and chemical stability of the specimens; SC specimens were post-cured using the LC-3DPrintBox unit (3D Systems, USA) for 30 min at an approximate irradiance of 300–400 mW/cm^2^, VS specimens were post-cured in the SprintRay ProCure 2 device (SprintRay, Inc., Los Angeles, CA, USA) under an irradiance of 2000 mW/cm^2^ for 10 min. For SM materials, cylindrical specimen designs (Ø3 × 12 mm) were created using same software programme. The CS and TC specimens were manufactured from prefabricated CAD/CAM blocks using a 5-axis milling machine (Ceramill Motion 2; Amann Girrbach AG, Koblach, Austria). The milling procedure was performed under constant water cooling to prevent overheating and preserve material integrity. Following milling, the resulting Ø3 × 12 mm cylindrical structures were sectioned into final dimensions of Ø3 × 4 mm using a precision cutting machine (Mecatome T180; Presi SA, Grenoble, France). All specimens were then subjected to wet grinding using silicon carbide abrasive papers (600–1200 grit, waterproof; Atlas, Saint-Gobain, Courbevoie, France) under running water to standardize the bonding surfaces. The specimens were randomly divided into three subgroups based on the surface treatment protocol to be applied: Primer only (PO), primer + phosphoric acid etching (PA), primer + airborne-particle abrasion (sandblasting) (PS). All surface treatment protocols (PO, PA, and PS) were applied exclusively to the bonding surfaces of the restorative specimens. The titanium surfaces in all groups were conditioned in a standardized manner involving airborne-particle abrasion and primer application, which is detailed in the following section. A visual summary of the distinct surface pretreatment steps applied to titanium substrates and restorative materials is presented in Figure 1.

Randomization was performed using a random number generator in spreadsheet software (Excel; Microsoft Corp., Redmond, WA, USA) to ensure unbiased allocation across groups (*n* = 10). A power analysis was conducted prior to specimen allocation to determine the minimum required sample size for statistical validity. Based on an alpha level of 0.05, a power of 0.80, and an estimated medium effect size (f = 0.42), it was calculated that a minimum of 10 specimens per subgroup would be sufficient.

A total of 40 titanium disks (Ø15.8 × 2 mm) were prepared by sectioning pre-milled custom titanium abutment blanks (Dentium Superline Pre-Milled Abutment; Dentium, Seoul, Republic of Korea) using the same precision cutting machine under water cooling. The surface properties of the specimens were designed to simulate clinical abutments. Therefore, all bonding surfaces were ground and polished using silicon carbide abrasive papers (600–1200 grit; Atlas, Saint-Gobain, France) under running water to eliminate irregularities and achieve a smooth surface finish. Prior to surface treatment, specimens were ultrasonically cleaned in distilled water for 5 min using an ultrasonic bath (JP-4820; Skymen Heatable, Shenzhen, China) and dried with oil-free compressed air to ensure surface cleanliness. To enhance micromechanical retention for subsequent bonding procedures, airborne-particle abrasion was applied to the bonding surfaces using 150 µm aluminum oxide particles (Cobra; Renfert GmbH, Hilzingen, Germany) at 0.2 MPa pressure, for 10 s, from a 10 mm distance using a sandblasting unit (Renfert Basic Solo; Renfert GmbH, Hilzingen, Germany). Following sandblasting, the surfaces were steam-cleaned for 10 s using a steam cleaner (Vap-8 Steamer; Zhermack GmbH, Badia Polesine, Italy). A universal adhesive primer containing 10-MDP was uniformly applied to the abraded bonding surface of each titanium specimen and allowed to air dry completely before cementation. No additional surface modifications were applied to the titanium surfaces. Together, these parameters reflect a standardized, evidence-based approach for conditioning titanium surfaces, ensuring that the experimental model closely mirrors established clinical protocols for adhesive cementation to titanium abutments [10,11]. To facilitate consistent specimen alignment during shear bond strength (SBS) testing, each titanium disk was embedded in autopolymerizing acrylic resin (Meliodent; Kulzer GmbH, Germany) using a Teflon mold (Ø20 × 30 mm).

All surface treatments were carried out under standardized laboratory conditions by the same operator. The sandblasting parameters used for titanium abutments and restorative specimens are summarized in Table 2. PO group: A universal adhesive primer was applied to the bonding surface of each specimen using a microbrush. The primer was left undisturbed for 60 s and then gently air-dried using oil-free compressed air. PS group: Specimens were subjected to airborne-particle abrasion using 50 μm aluminum oxide particles at 0.2 MPa pressure, applied for 10 s from a 10 mm distance. After sandblasting, the surfaces were cleaned using oil-free air, and the same universal primer was applied for 60 s. PA group: Bonding surfaces were treated with 35–40% phosphoric acid gel (DeTrey Conditioner 36; Dentsply Sirona, USA) for 30 s, rinsed thoroughly with water, and dried with oil-free air. Then, the 10-MDP based primer was applied for 60 s and air-dried. All procedures were performed according to the manufacturer’s recommendations for each chemical agent. Treated specimens were then stored in a light-protected, dry environment until the cementation step.

A dual-cure resin cement (G-CEM LinkForce; GC Corporation, Japan) was used for bonding the restorative specimens to the titanium substrates. For each specimen, a thin and uniform layer of resin cement was applied to both the surface of the treated restorative material and the preconditioned titanium disk using a disposable microbrush. The restorative specimen was then carefully positioned on the titanium surface, and manual finger pressure was applied for approximately 60 s to ensure proper adaptation. After positioning, excess cement was gently removed using a disposable applicator tip. Light polymerization was performed from the center using an LED curing unit (Elipar S10; 3M ESPE, St. Paul, MN, USA) at 1200 mW/cm^2^ for 60 s, ensuring complete curing of the marginal areas. Following polymerization, the specimens were stored in distilled water at 37 °C for 24 h in accordance with ISO TR 11405:2015 guidelines [19] short-term storage prior to bond strength testing. This storage condition was selected based on literature supporting its use as a standardized short-term screening protocol, rather than as a predictor of long-term clinical durability [20,21,22]. All cementation procedures were carried out under controlled temperature and humidity conditions, and care was taken to avoid contamination of the bonding surfaces throughout the process.

**Figure 2:**
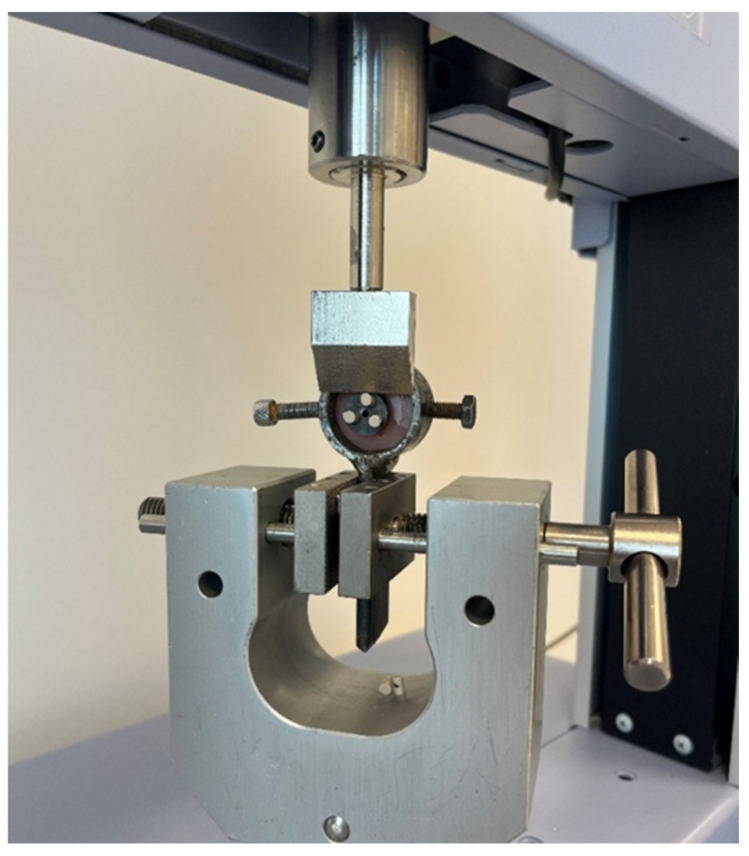
Application of shear bond strength test using a universal testing device.

SBS testing was performed using a universal testing machine (AGX-S; Shimadzu Corporation, Kyoto, Japan). The testing protocol was conducted in accordance with ISO 10477:2020 guidelines [23] for evaluating the bond strength of dental restorative materials. Each titanium–restorative specimen assembly was securely mounted to the testing jig using the threaded screw embedded in the acrylic resin base. A knife-edged loading blade was aligned perpendicular (90°) to the adhesive interface and positioned as close as possible to the bonding area to ensure an accurate shear force application (Figure 2).

The test was conducted at a crosshead speed of 1 mm/min, and the maximum force at failure was recorded in Newtons (N). The bonding area was calculated based on the standardized specimen diameter (Ø3 mm), and SBS values were expressed in megapascals (MPa) using the following Equation (1):


(1) $$SBS\;\left(MPa\right)=\frac{Failure\;load\;\left(N\right)}{Bonding\;area\;\left({mm}^{2}\right)}$$

After debonding, all failure loads were tabulated, and the corresponding SBS values were computed for each specimen prior to statistical analysis.

All data obtained from SBS tests were statistically analyzed using SPSS software (Version 20.0; IBM Corp., Chicago, IL, USA). The level of significance was set at α = 0.05 for all tests, and results were reported with corresponding *p*-values. The Shapiro–Wilk test was first used to evaluate the normality of the SBS data distribution within each group. Subsequently, the Levene’s test was applied to assess the homogeneity of variances. Given that the data met assumptions of normality and homogeneity, a two-way analysis of variance (ANOVA) was conducted to evaluate the effects of restorative material type and surface treatment method on SBS. Where significant interactions or main effects were observed, post-hoc comparisons were performed using the Tamhane’s T2 post hoc test. In addition, the distribution of failure modes was categorized as adhesive; failure at the material–cement interface, cohesive; failure within the material, and mixed; combination of adhesive and cohesive failures.

## 3. Results

### 3.1. Normality and Homogeneity of Variance

According to the Shapiro–Wilk test, all groups demonstrated a normal distribution (*p* > 0.05), indicating that the assumption of normality required for parametric testing was met. Based on this, a two-way ANOVA was performed to evaluate the effects of the factors and their interaction on SBS (Table 3). However, normal distribution alone does not guarantee equal variances across groups. Therefore, Levene’s test was additionally conducted to assess the homogeneity of variances, and it revealed a significant result (*p* < 0.05), indicating that this assumption was violated (Table 4). Although two-way ANOVA is considered relatively robust to moderate variance heterogeneity in balanced designs, a post hoc procedure that does not assume equal variances was chosen to ensure more reliable pairwise comparisons. Consequently, Tamhane’s T2 test was used for multiple comparisons between groups.

### 3.2. Surface Morphology Analysis (SEM Evaluation)

**Figure 3:**
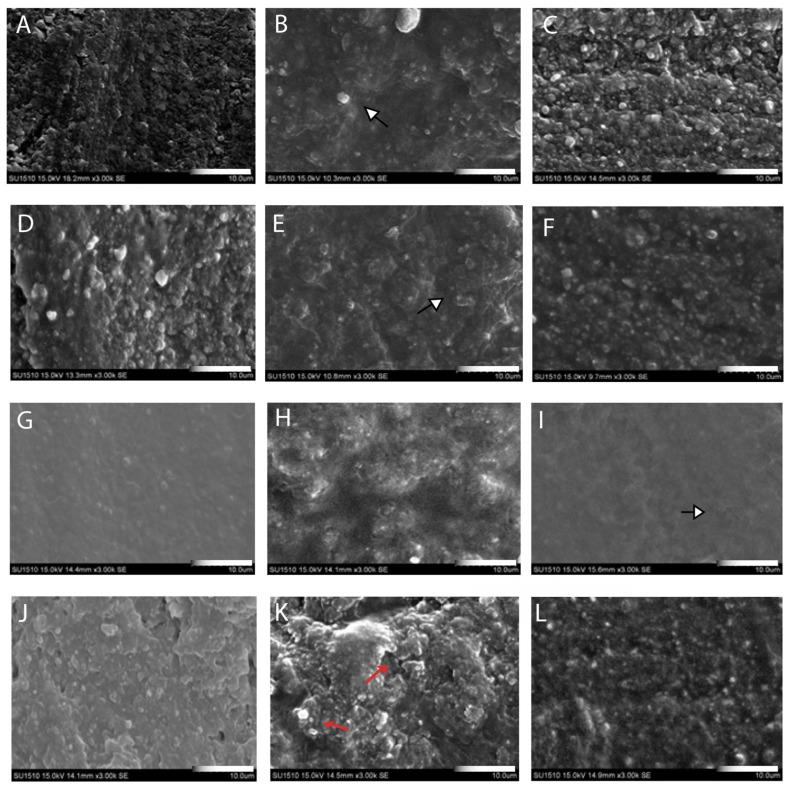
Representative scanning electron microscopy (SEM) (Jeol, Tokyo, Japan) images (original magnification ×3000) illustrating the surface morphology of resin-based CAD/CAM materials after different surface treatment protocols. Images correspond to Tetric CAD (**A**–**C**), Saremco Crowntec (**D**–**F**), Cerasmart (**G**–**I**), and VarseoSmile Crown Plus (**J**–**L**). For each material, panels show primer only (PO), primer combined with airborne-particle abrasion (PS), and primer combined with phosphoric acid etching (PA), respectively. White arrows indicate representative regions of moderate surface roughness in Saremco Crowntec (panel **E**) and mild surface roughness in Tetric CAD (panel **B**) following airborne-particle abrasion, as well as relatively smooth surface morphology observed in phosphoric acid–treated groups (panel **I**). Red arrows highlight pronounced surface irregularities, particularly evident in VarseoSmile Crown Plus (panel **K**) specimens after airborne-particle abrasion.

SEM evaluation demonstrated that surface morphologies varied depending on the material type and surface treatment applied. The PO groups exhibited smooth and relatively homogeneous surfaces with minimal irregularities. This surface condition was observed in all materials, particularly CS and TC specimens exhibiting low surface roughness under this condition. In the PS groups, all materials presented rougher and more irregular surface textures. These specimens exhibited define microporosities, grooves, and increased surface area. VS specimens showed the most pronounced surface alterations following sandblasting. SC and TC specimens also exhibited increased surface topography, while CS presented a granular and moderately rough surface. The PA groups showed minimal topographical changes compared to the PS groups. In materials such as CS and VS, surface morphology remained relatively smooth with low porosity following acid application (Figure 3).

### 3.3. Shear Bond Strength (SBS) Analysis

Two-way ANOVA results showed that both the type of restorative material (*p* < 0.001, η^2^ = 0.994) and surface treatment (*p* < 0.001, η^2^ = 0.983) had statistically significant effects on SBS, with a significant interaction between these two factors (*p* < 0.001, η^2^ = 0.959) (Table 5).

Simple effects analysis showed that the magnitude of the surface treatment effect on SBS differed across materials, with partial eta squared (η^2^) values of 0.991 for SC, 0.989 for TC, 0.960 for CS, and 0.924 for VS. Among all material-treatment combinations, the highest mean SBS value was observed in the PS_SC group (18.48 ± 0.13 MPa), followed by PA_SC (15.05 ± 0.07 MPa) and PS_VS (14.15 ± 0.18 MPa). The lowest SBS values were recorded in PO_CS (8.07 ± 0.17 MPa) and PO_TC (8.29 ± 0.24 MPa). For each material, PS application resulted in the highest SBS values, while PO groups consistently exhibited the lowest. Notably, CS showed the smallest difference among surface treatments, with all SBS values remaining below 9.4 MPa. In contrast, SC demonstrated the greatest difference, ranging from 12.38 MPa (PO) to 18.48 MPa (PS). Tamhane’s post hoc analysis revealed statistically significant differences between multiple group pairs, especially between PO and PS groups for the same materials, confirming the effect of surface conditioning protocols on bond performance (Table 6). The raw shear bond strength (SBS) values used for statistical analysis are provided in the Supplementary Materials (Table S1).

### 3.4. Failure Mode Distribution

**Figure 4:**
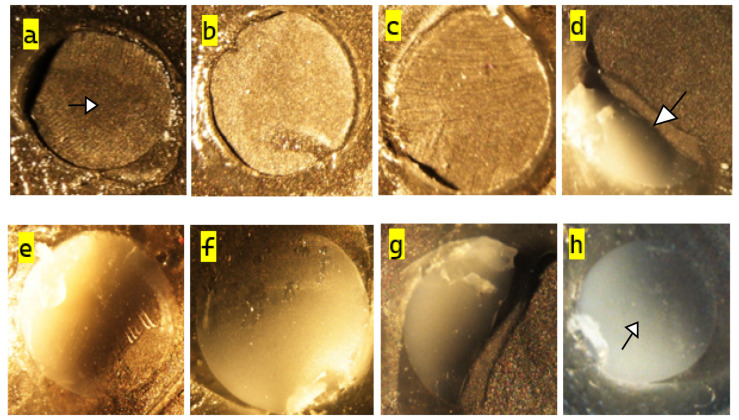
Stereomicroscopic images of titanium abutment specimens after shear bond strength testing (original magnification ×250). Tetric CAD showing adhesive failure (**a**,**b**); Cerasmart with adhesive failure (**c**); Saremco Crowntec showing mixed failure (**d**,**e**); VarseoSmile Crown Plus with mixed failure (**f**,**g**) and cohesive failure (**h**). Arrows indicate representative regions illustrating adhesive (**a**), mixed (**d**), and cohesive (**h**) failure modes.

In the PO groups, adhesive failures predominated in all materials. TC and CS specimens exhibited exclusively adhesive failures (10/10), whereas SC and VS groups showed a combination of adhesive (7/10) and cohesive (3/10) failures, with no mixed fractures observed. In the PS groups, a shift toward mixed failures was observed. VS specimens demonstrated the highest rate of mixed fractures (6/10), followed by SC (5/10). TC specimens exhibited predominantly adhesive failures (8/10), and CS maintained a high proportion of adhesive failures (9/10), with only one mixed failure. In the PA groups, adhesive fractures remained the most prevalent failure type across all materials, particularly in CS (10/10) and TC (9/10). Mixed fractures were observed in SC (3/10) and VS (5/10), with a single cohesive failure reported in the latter (Table 7), (Figure 4).

## 4. Discussion

In this study, the null hypothesis stated that neither the restorative material type nor the applied surface treatment would significantly affect the SBS. However, the results obtained clearly contradicted this hypothesis. Both the type of restorative material and the surface conditioning protocol significantly influenced the SBS values (*p* < 0.001), and a significant interaction was observed between these two factors as well (*p* < 0.001). Therefore, the null hypothesis was rejected.

When the SBS values were compared, AM materials demonstrated consistently higher mean bond strength than SM ones within the specific materials and processing conditions investigated. Among all combinations, PS_SC exhibited the highest SBS (18.48 ± 0.13 MPa), while the lowest values were observed in PO_CS (8.07 ± 0.17 MPa). Across all restorative materials, PS application yielded the most favorable SBS outcomes, followed by PA, and finally PO groups. These findings indicate that both material composition and mechanical surface modification substantially impact adhesive bonding efficiency.

A notable similarity was observed between the results of the present study and those reported by Küçükekenci et al. [17], particularly in terms of the influence of manufacturing techniques on bond strength. In both studies, additively manufactured (AM) resin materials consistently outperformed their subtractively manufactured (SM) counterparts in terms of SBS. Specifically, Küçükekenci et al. reported SBS values ranging from 14.59 to 14.94 MPa for AM specimens bonded to titanium abutments, whereas SM specimens exhibited significantly lower values, ranging from 5.88 to 9.57 MPa. In the present study, a similar trend was evident, as the AM group achieved a mean SBS of 14.35 ± 1.53 MPa, markedly higher than the 9.78 ± 1.44 MPa observed in the SM group (*p* < 0.05).

The observed differences in SBS values between SM and AM methods can be partially explained by the inherent characteristics of their respective fabrication processes [24,25]. However, it should be acknowledged that, in the present study, the two AM materials were fabricated on different printing and post-curing systems, each following its own manufacturer-specific protocol. Thus, the superior bond strength observed for AM groups likely reflects a combined effect of manufacturing mode (additive vs. subtractive), individual material composition, and system-specific processing parameters, rather than the manufacturing method alone [2,26].

Although the AM materials tested in this study exhibited higher initial bond strength to titanium abutments than the SM counterparts, this advantage must be interpreted in the context of their overall material properties. Previous investigations have indicated that many AM resins tend to show lower flexural strength, fracture toughness, and wear resistance, as well as higher polymerization shrinkage and water sorption, than certain high-performance milled composites [15,18]. These characteristics may generate interfacial stresses, microcracking, and hydrolytic degradation over time, potentially offsetting the benefits of improved micromechanical retention under clinical loading and aging conditions [2,24]. Consequently, superior short-term SBS values for AM materials should not be equated with superior long-term clinical performance, and material selection should balance bonding behavior with mechanical and aging properties [20,22].

Subtractive resins are produced using controlled industrial conditions that involve elevated temperature and pressure, which promotes a homogeneous internal structure and reduce potential defects. In contrast, additively manufactured materials are formed through layer-by-layer polymerization of resin, which may inherently introduce interfacial inconsistencies or microvoids between layers [17,24,27]. Although such features might be considered structural weaknesses, they can facilitate resin cement infiltration and improve micromechanical retention, ultimately enhancing the adhesive bond to the abutment surface. These findings are consistent with those of Küçükekenci et al. [17] but extend the current understanding by analyzing how different surface treatment protocols interact with the internal architecture of AM and SM resins.

Within the AM group, SC and VS also showed some differences in SBS and surface morphology, which may be related not only to their distinct resin formulations but also to the different printing and post-curing devices used. These further underlines that “additive manufacturing” represents a heterogeneous category, and system-specific factors should be considered when interpreting the results. Such an approach provides a more detailed perspective on the micromechanical and chemical factors influencing bonding performance and may contribute to the refinement of clinical cementation strategies, within the limitations of an in vitro, short-term study design. Surface pretreatments prior to resin cement application are essential for establishing effective micromechanical retention [28]. Numerous studies have demonstrated that mechanical surface roughening typically results in higher bond strength than chemical treatments alone [25,29,30]. Airborne-particle abrasion enhances both surface roughness and surface energy by generating microstructural irregularities, which in turn promote improved wetting and penetration of the resin cement—critical factors for durable bonding [31,32]. These effects were clearly visible in SEM images from present study, where sandblasted specimens displayed a markedly irregular topography characterized by microgrooves and microporosities. Such morphological changes were particularly evident in the PS group, supporting the higher SBS values observed and confirming enhanced micromechanical interlocking at the resin-material interface. However, because surface roughness was not quantified using profilometry or 3D texture analysis, these correlations remain qualitative, and future studies should incorporate Ra/Rz measurements or image-based metrics to more precisely relate topographical changes to bond strength.

Beyond increasing surface texture, sandblasting also eliminates contaminants—such as saliva—that may be introduced during clinical try-in, thus providing a cleaner bonding substrate [33]. Multiple studies have sought to optimize the parameters of this process, indicating that pressures between 1 and 3 bar (0.1 MPa ≈ 1 bar) and durations ranging from 5 to 60 s are generally effective, although the ideal values may vary depending on the composite material [34,35]. Nevertheless, when parameters are not properly calibrated, SEM analysis reveals microcracks and interfacial damage within the resin matrix or filler–resin interface, which can jeopardize long-term restoration success [36,37]. In a recent investigation, Dederichs et al. [38] examined the effect of sandblasting pressure on 3D-printed permanent crown resin using 50 μm aluminum oxide at pressures of 1, 2, and 3 bar. The study reported mean bond strengths of 25.57 ± 7.04 MPa, 28.14 ± 6.35 MPa, and 30.15 ± 6.46 MPa, respectively. Notably, SEM evaluation also revealed that higher pressures increased the risk of subsurface microfractures. Based on these findings, the present study applied sandblasting at 0.2 MPa for 10 s, balancing bond strength with surface integrity.

The failure mode distribution further supports the SBS and SEM findings. Groups with predominantly adhesive failures, particularly CS and TC under PO and PA conditions, exhibited lower bond strength, suggesting a weak interfacial attachment that fractured mainly at the material–cement interface. In contrast, mechanically treated AM groups (especially PS_SC and PS_VS) showed a higher proportion of mixed and occasional cohesive failures, with stereomicroscopic analysis revealing residual cement and fractured restorative material remaining on both the titanium and resin surfaces. This shift from purely adhesive to mixed/cohesive failures is consistent with a stronger and more integrated bond, in which the applied stresses are partially transferred into the bulk of the restorative material rather than being concentrated solely at the interface. These observations indicate that both surface roughening and material microstructure influence not only the magnitude of SBS, but also the locus of failure.

Complementing the mechanical treatments, chemical surface conditioning also contributes significantly to adhesion effectiveness. With the decreasing reliance on silane-only agents and the broader adoption of universal primers—containing both silane and functional monomers like 10-MDP—this study focused on the effects of universal primer application. Silane molecules possess bifunctional groups that enable interaction with both the inorganic fillers and the organic resin matrix, facilitating strong chemical adhesion through siloxane bonds and enhancing wettability for better monomer infiltration. This dual mechanism is especially advantageous in hybrid materials [39,40]. The inclusion of 10-MDP within universal primers, such as Monobond Plus, has been reported to further amplify adhesion performance. The phosphate groups in 10-MDP can chemically bond to metal oxides and ceramic phases, while the methacrylate groups copolymerize with the resin cement matrix. In 3D-printed materials—which often exhibit higher organic content and may retain unreacted monomers post-polymerization—this interaction has been suggested to become even more critical [18,26]. Recent studies suggest that 10-MDP may demonstrate more pronounced effects in such materials compared to milled alternatives, although further evidence is required to validate this hypothesis [25,41]. Consistent with these reports, the present study demonstrated that SBS values in the PO_SC (12.38 ± 0.38 MPa) and PO_VS (12.72 ± 0.13 MPa) groups were statistically significantly higher than those in the PO_CS (8.07 ± 0.17 MPa) and PO_TC (8.29 ± 0.24 MPa) groups, which may indicate a more favorable interaction between 10-MDP–containing primers and the resin-rich matrix of AM materials. However, it should be emphasized that this mechanism was not directly confirmed in the present study, as no surface analytical techniques (e.g., XPS, EDS, FTIR) were employed; therefore, the proposed 10-MDP–TiO_2_ interaction should be interpreted as a plausible explanation supported by previous literature rather than a finding demonstrated here.

The findings of this study indicate that PA groups exhibited lower SBS values in comparison to PS groups. Although mechanical surface treatments are known to enhance bonding by increasing surface irregularities and roughness, their application should follow material-specific protocols due to the risk of surface deformation and material loss [38,42]. Acid-based surface treatments can generate a favorable surface texture and roughness, particularly on ceramic materials, by creating a topography that supports micromechanical interlocking. In feldspathic ceramics, hydrofluoric acid (HF) reacts chemically with silica, forming hexafluorosilicates that contribute to microretention [43]. However, due to its potential for inducing cellular toxicity and associated risks, the utilisation of HF for intraoral use may be contraindicated [44,45]. Additionally, its application on hybrid ceramics, particularly resin-based nanoceramics, is not recommended. Studies have demonstrated that HF does not produce sufficient surface roughness in such materials and may even impair bond strength by softening the resin matrix or dissolving exposed glass fillers, ultimately compromising adhesion performance [46,47,48].

Phosphoric acid, although less aggressive, is considered a safer and more practical alternative for chairside procedures. Nonetheless, its limited etching capacity often renders it more suitable as a surface cleaning agent rather than a true conditioner capable of forming retentive microstructures [45,49]. In the present study, SBS values in the PA_VS (13.31 ± 0.20 MPa) and PA_SC (15.05 ± 0.07 MPa) groups were significantly higher than those in the PA_CS (8.96 ± 0.09 MPa) and PA_TC (10.20 ± 0.18 MPa) groups. Although phosphoric acid is not typically expected to promote micromechanical bonding, the results suggest that, when applied to compatible materials, it may still provide adequate bond strength. Nevertheless, in SM materials characterized by greater structural density, the role of phosphoric acid in facilitating adhesion appears to be minimal.

Contrary to the findings of Donmez et al. [50], who reported significantly higher SBS values for SM polymer-infiltrated ceramic network (PICN) materials compared to AM_SC and AM_VS when bonded to titanium, the present study demonstrated an opposite trend. This discrepancy may be attributed to a combination of methodological and material-related differences between the studies. Additionally, variations in the chemical composition of the PICN materials and the types of resin cements used could have contributed to the contrasting outcomes by influencing the interfacial interaction and polymer infiltration capacity. In the present study, SEM analysis revealed that AM materials exhibited more porous surface morphologies, indicating a greater potential for micromechanical interlocking compared to SM materials, which possess a denser microstructure due to their higher ceramic content. These structural differences likely contributed to the higher SBS values observed in AM groups, particularly following airborne-particle abrasion. In contrast, the compact nature of SM materials may necessitate alternative or more intensive surface treatment protocols to optimize adhesive performance. This interpretation is supported by the observation that the lowest SBS values were recorded in the SM_CS groups [PO (8.07 ± 0.17 MPa), PA (8.96 ± 0.09 MPa), PS (9.37 ± 0.01 MPa)]. Therefore, rather than highlighting the efficacy of current pretreatment strategies, the findings underscore the need for further optimization of surface modification protocols tailored specifically to material microstructure. It should also be emphasized that, in the present study, a single airborne-particle abrasion protocol (50 µm Al_2_O_3_ at 0.2 MPa for 10 s) was deliberately applied to all resrorative materials to enable comparison under standardized conditions and was not intended to identify the optimal pressure for each composite. Consequently, the present results reflect how AM and SM materials respond to this standardized protocol, and further experimental work is required to determine material-specific optimal sandblasting pressures, particularly for SM composites.

This in vitro study presents several limitations that must be acknowledged. All additively manufactured specimens were produced using a standardized layer thickness and printing angle based on manufacturer recommendations; thus, variations in these parameters may yield different outcomes. In addition, the two AM materials were printed on different 3D-printing and post-curing systems, each with distinct light intensities and curing protocols. Consequently, the higher SBS values observed for AM materials in this study cannot be attributed solely to the manufacturing mode itself, but may also be influenced by material-specific formulation and system-dependent processing conditions. Only one type of resin cement, acid, sandblasting protocol, and adhesive agent was used, limiting comparative evaluation of alternative materials and methods. In addition, wettability was not assessed by contact angle measurements, which limits the ability to quantitatively relate surface roughness and surface energy to bonding performance. In particular, a single airborne-particle abrasion protocol was intentionally applied to all groups to standardize surface treatment and allow comparison under identical conditions; as a result, the present data cannot identify material-specific optimal sandblasting pressures, especially for SM composites. In addition, surface roughness was not quantified (e.g., Ra, Rz), which limits direct numerical correlation between topography and SBS. Furthermore, the study focused solely on initial SBS values without applying thermomechanical aging protocols, which restricts predictions about long-term clinical performance. Factors such as thermal cycling, pH fluctuations, and fatigue loading—although not assessed here—are known to influence resin bonding durability. In addition, the 24-h water storage at 37 °C represents only short-term conditioning of the adhesive interface. Longer storage periods and/or artificial aging procedures are required to more accurately simulate the degradation that occurs in vivo. Future studies incorporating a wider range of materials, manufacturing parameters, and artificial aging protocols are necessary to better simulate clinical conditions and validate the adhesive performance of both milled and 3D-printed CAD/CAM restorative materials.

## 5. Conclusions

Considering the outcomes of this in vitro, short-term study, the following conclusions are presented:

1. Surface treatment plays a critical role in bonding performance. Airborne-particle abrasion significantly enhances SBS by increasing surface roughness and promoting micromechanical retention.
2. Additively manufactured resin-based CAD/CAM materials tested in the present study showed higher initial bond strength to titanium abutments than the evaluated subtractively manufactured materials, which may be related to their more favorable surface morphology and/or system-specific processing parameters. However, these results are based on 24-h water storage and should not be interpreted as definitive evidence of superior long-term clinical performance, nor should they be generalized to all AM and SM systems.
3. Combined mechanical and chemical surface treatments provide better adhesion than chemical treatment alone. The use of 10-MDP–containing universal primers is effective, particularly when preceded by surface roughening, within the short-term conditions tested.
4. Each restorative material responds differently to surface treatments, indicating the necessity of material-specific bonding protocols rather than a universal approach.
5. Subtractive materials, due to their dense microstructure, may require more aggressive or alternative surface treatments to achieve higher initial bond strength. Further studies, including aging protocols are required before definitive clinical recommendations can be made.
6. Failure modes varied according to surface treatment and material, with mechanically treated groups showing more mixed and cohesive failures, suggesting stronger interfacial bonding.

## Figures and Tables

**Table 1:** Chemical composition of the materials used in the present study.

Material	Material Type	Composition	Manufacturer
Dentium Superline Pre-Milled Abutment	Titanium	Grade V Titanium;Ti-6AL-4V	Dentium, Seoul, Republic of Korea
Crowntec	3D-printed permanent composite resin material	4,4-isopropylphenol, ethoxylated and esterified with 2-methylprop-2-enoic acid, silanized dental glass, pyrogenic silica (SiO_2_), initiators. Total inorganic filler content: 30–50% (by weight)	Saremco Dental AG, Rebstein, Switzerland
VarseoSmile Crown Plus	3D-printed permanent hybrid composite resin material	Esterification products of 4,4-isopropylphenol, ethoxylated and 2-methylprop-2-enoic acid, silanized dental glass, methyl benzoylformate, diphenyl (2,4,6-trimethylbenzoyl) phosphine oxide. Total inorganic filler content: 30–50% (by weight)	BEGO, Bremen, Germany
Tetric CAD	Composite Resin Material	Cross-linked dimethacrylate matrix containing 80% (by weight) nanoparticles	Ivoclar-Vivadent AG, Schaan, Liechtenstein
Cerasmart	Nano-ceramic resin composite material	UDMA, Bis-MEPP, dimethacrylate, 71% (by weight) silica (40 nm), barium-silica nanoparticles	GC Corporation, Tokyo, Japan
G-CEM LinkForce	Dual-cure adhesive resin cement	Resin-based composite cement: A: Bis-GMA, UDMA, DMA, barium silica, initiator, pigment B: Bis-MEPP, UDMA, DMA, barium silica, initiator	GC Corporation, Tokyo, Japan
Monobond Plus	Universal Primer	Ethanol, 3-trimethoxysilylpropyl methacrylate (silane), methacrylated phosphoric acid ester (10-MDP), and disulfide acrylate	Ivoclar-Vivadent AG, Schaan, Liechtenstein
DeTrey Conditioner 36	Phosphoric Acid	Phosphoric acid, highly dispersed silicon dioxide, detergent, pigment, water	Dentsply Sirona, Charlotte, NC, USA

**Table 2:** Sandblasting parameters applied to titanium and restorative materials.

Substrate	Abrasive	Particle Size (µm)	Pressure (MPa)	Distance (mm)	Duration (s)
Titanium abutment (Grade V)	Aluminum oxide (Al_2_O_3_)	150	0.2	10	10
Restorative Materials	Aluminum oxide (Al_2_O_3_)	50	0.2	10	10

**Table 3:** Shapiro-Wilk test results.

Group	Shapiro-Wilk Statistics	df	Sig.
TC_PO	0.908	10	0.270
TC_PS	0.981	10	0.971
TC_PA	0.909	10	0.273
SC_PO	0.940	10	0.555
SC_PS	0.896	10	0.197
SC_PA	0.897	10	0.203
CS_PO	0.952	10	0.690
CS_PS	0.918	10	0.340
CS_PA	0.942	10	0.581
VS_PO	0.913	10	0.306
VS_PS	0.919	10	0.346
VS_PA	0.874	10	0.110

*p*-values derived from Shapiro-Wilk test. Significance threshold set at 0.05.

**Table 4:** Levene’s Homogeneity test results.

F	df1	df2	Sig.
4.780	11	108	0.000 *

* *p*-values derived from Levene’s test. Significance threshold set at 0.05.

**Table 5:** Two-Way ANOVA results.

	Type III Sum of Squares	df	F	*p*	Partial Eta Squared
Material Type	738.080	3	6042.079	0.000 *	0.994
Surface Treatment	258.633	2	3175.832	0.000 *	0.983
Material Type & Surface Treatment	103.567	6	423.911	0.000 *	0.959
Error	4.398	108			
Total	18,581.537	120			
Corrected Total	1104.677	119			

* p-values less than 0.001 indicate statistically significant differences.

**Table 6:** Descriptive statistics of shear bond strength (MPa) to titanium according to material type and surface treatment, including mean values, standard deviations, and 95% confidence intervals (CI).

	PO	PS	PA
Material	Mean ± Standard Deviation (95%CI) (MPa)	Mean ± Standard Deviation (95%CI) (MPa)	Mean ± Standard Deviation (95%CI) (MPa)
TC	8.29 (±0.24) ^A^ (8.13–8.49)	13.80 (±0.30) ^G^ (13.57–14.03)	10.20 (±0.18) ^D^ (10.07–10.34)
SC	12.38 (±0.38) ^E^ (12.10–12.67)	18.48 (±0.13) ^I^ (18.38–18.58)	15.05 (±0.07) ^H^ (15.01–15.11)
CS	8.07 (±0.17) ^A^ (7.94–8.20)	9.37 (±0.01) ^C^ (9.36–9.38)	8.96 (±0.09) ^B^ (8.89–9.03)
VS	12.72(±0.13) ^E^ (12.61–12.81)	14.15 (±0.17) ^G^ (14.02–14.28)	13.31 (±0.20) ^F^ (13.16–13.46)
Total	10.36 (±2.23) (9.65–11.07)	13.95 (±3.26) (12.91–14.99)	11.88 (±2.45) (11.10–12.66)

Different uppercase letters within the same row or column indicate statistically significant differences (*p* < 0.05), as determined by Tamhane post-hoc multiple comparison test.

**Table 7:** Distribution of failure modes (adhesive, cohesive, mixed) by material type and surface treatment, presented as frequency (n) and percentage (%).

Material Type	PO			PS			PA			Total		
	Adh	Coh	Mix	Adh	Coh	Mix	Adh	Coh	Mix	Adh	Coh	Mix
TC	10 (%100)	0 (%0)	0 (%0)	8 (%80)	0 (%0)	2 (%20)	9 (%90)	0 (%0)	1 (%10)	27 (%90)	0 (%0)	3 (%10)
SC	7 (%70)	0 (%0)	3 (%30)	4 (%40)	1 (%10)	5 (%50)	7 (%70)	0 (%0)	3 (%30)	18 (%60)	1 (%3)	11 (%37)
CS	10 (%100)	0 (%0)	0 (%0)	9 (%90)	0 (%0)	1 (%10)	10 (%100)	0 (%0)	0 (%0)	29 (%97)	0 (%0)	1 (%3)
VS	7 (%70)	0 (%0)	3 (%30)	2 (%20)	2 (%20)	6 (%60)	4 (%40)	1 (%10)	5 (%50)	13 (%43)	3 (%10)	14 (%47)
Total	34 (%85)	0 (%0)	6 (%15)	23 (%58)	3 (%7)	14 (%35)	30 (%75)	1 (%2)	9 (%23)	107 (%76)	4 (%4)	29 (%20)

## Data Availability

The original contributions presented in this study are included in the article. Further inquiries can be directed to the corresponding author.

## Supplementary Materials

The following supporting information can be downloaded at: https://www.mdpi.com/article/10.3390/polym18010056/s1, Table S1: The raw shear bond strength (SBS) data for all experimental groups.

## Abbreviations

The following abbreviations are used in this manuscript:

AM	Additive manufacturing
SM	Subtractive manufacturing
SC	Saremco Crowntec
VS	VarseoSmile Crown Plus
TC	Tetric CAD
CS	Cerasmart
PO	Primer only
PA	Primer + phosphoric acid etching
PS	Primer + airborne-particle abrasion
SBS	Shear bond strength
PMMA	Polymethyl methacrylate
MMA	Methyl methacrylate
MDP	10-Methacryloyloxydecyl dihydrogen phosphate
UDMA	Urethane dimethacrylate
HEMA	2-Hydroxyethyl methacrylate
TEGDMA	Triethylene glycol dimethacrylate
Bis-GMA	Bisphenol A-glycidyl methacrylate
DMA	Dimethacrylate
