# Supplementary material for: Comparison of the Bond Strength to Titanium of Resin-Based Materials Fabricated by Additive and Subtractive Manufacturing Methods
Source: Polymers (Basel). 2025 Dec 24;18(1):56. doi: 10.3390/polym18010056 (PMC12787806; doi:10.3390/polym18010056)
Supplement: Supplementary file 1 [file polymers-18-00056-s001.zip › polymers-4044597-supplementary.pdf]

TC_PO	TC_PS	TC_PA	CS_PO	CS_PS	CS_PA	SC_PO	SC_PS	SC_PA
8.505897	14.32113	10.13467	7.795389	9.366033	9.114577	12.45563	18.3562	15.07346
8.044237	13.8953	10.30338	8.11082	9.364028	8.971531	12.62456	18.32145	14.95801
8.12847	13.74889	10.43441	8.296326	9.367944	8.830964	12.47511	18.56602	15.06037
8.109374	13.57532	10.14948	8.122269	9.370377	9.066733	12.8559	18.61697	15.07098
8.206761	13.51475	9.976007	8.104871	9.360797	8.978686	11.72534	18.39014	15.07895
8.050279	13.6995	10.00099	8.125504	9.36135	8.993802	12.10776	18.68431	15.11452
8.516832	13.83631	9.974103	8.229061	9.36799	8.845979	11.74377	18.66361	14.91484
8.2388	14.34158	10.25108	8.235878	9.388969	8.847926	12.49952	18.4464	15.04497
8.804389	13.39285	10.43164	7.82193	9.359896	8.977929	12.8316	18.41477	15.17105
8.463321	13.68006	10.43516	7.871671	9.387916	9.000472	12.51168	18.36374	15.09691

VS_PO	VS_PS	VS_PA
12.77391	14.31415	13.16929
12.70283	13.86899	13.6014
12.81419	14.10363	13.58289
12.57825	14.39089	13.41185
12.60681	14.20568	13.00005
12.65917	14.00187	13.17446
12.50523	14.23965	13.42256
12.89298	13.97134	13.31524
12.92077	14.04599	13.03259
12.65012	14.36702	13.38967
